# Exploiting Fine-Grained Subcarrier Information for Device-Free Localization in Wireless Sensor Networks

**DOI:** 10.3390/s18093110

**Published:** 2018-09-14

**Authors:** Yan Guo, Dongping Yu, Ning Li

**Affiliations:** College of Communications Engineering, Army Engineering University of PLA, Nanjing 210007, China; guoyan_1029@sina.com (Y.G.); lining_friend@sina.com (N.L.)

**Keywords:** device-free localization, wireless sensor networks, compressive sensing, frequency diversity, joint sparse recovery

## Abstract

Device-free localization (DFL) that aims to localize targets without carrying any electronic devices is addressed as an emerging and promising research topic. DFL techniques estimate the locations of transceiver-free targets by analyzing their shadowing effects on the radio signals that travel through the area of interest. Recently, compressive sensing (CS) theory has been applied in DFL to reduce the number of measurements by exploiting the inherent spatial sparsity of target locations. In this paper, we propose a novel CS-based multi-target DFL method to leverage the frequency diversity of fine-grained subcarrier information. Specifically, we build the dictionaries of multiple channels based on the saddle surface model and formulate the multi-target DFL as a joint sparse recovery problem. To estimate the location vector, an iterative location vector estimation algorithm is developed under the multitask Bayesian compressive sensing (MBCS) framework. Compared with the state-of-the-art CS-based multi-target DFL approaches, simulation results validate the superiority of the proposed algorithm.

## 1. Introduction

Location information is an important ingredient for many location-based services (LBS) [1]. With the increasing demand of LBS, target localization technique has attracted considerable attention. In the last decade, extensive research works on target localization have been carried out by the scientific community. However, most previous approaches are device-based, which require targets to carry electronic devices or tags [2]. Different from device-based solutions, device-free localization (DFL) [3,4,5] do not have the requirement of equipping targets with any electronic devices, nor needs targets to participate actively in the localization process. It is an attractive and challenging technique and can significantly improve the usability and practicality of many localization applications, such as intruder detection, security surveillance, elder monitoring, emergency rescue, etc. [6].

The DFL technique can empower existing wireless infrastructures with the ability of location awareness while, at the same time, do not disturb the normal communication tasks [7]. In DFL, the location information of transceiver-free targets is estimated by analyzing the target-induced perturbations in the radio frequency (RF) field. In the literature, based on how to utilize the radio signal measurement information, the DFL approaches can be categorized into three types: geometry-based approaches, fingerprinting-based approaches, and radio tomographic imaging (RTI)-based approaches [8]. The geometry-based DFL approach utilizes the geometry information of shadowed links for location estimation [9,10,11], which needs a prior knowledge of the deployment of wireless links and has a low localization accuracy. The fingerprinting-based DFL approach can improve the localization accuracy [12,13], but a labor-intensive and time-consuming site survey is required to build and retraining the radio map to maintain fingerprint-location matching. The RTI system can locate targets accurately based on the principle of computed tomography (CT) [14,15]. However, a sufficient number of wireless links is required to cover the monitoring area, which may incur high energy consumption and high hardware cost.

Recent works have shown the potential of applying the compressive sensing (CS) [16,17] technique in target localization. CS is a promising technique for signal processing, which asserts that far fewer samples than Nyquist rate will suffice for recovering sparse or compressible signals. SLMLU [18] translates target localization problem into CS problem, and utilizes an LU decomposition preprocessing to make the measurement matrix meet restricted isometry property (RIP) [19]. Compared to the traditional DFL approaches, the CS-based DFL approach can achieve much higher localization accuracy with much less measurements (or wireless links). LCS [20] is a representative work, which estimates the sparse location vector based on the greedy matching pursuit (GMP) algorithm and has proven that the product of dictionary obeys RIP with high probability. As another CS-based DFL approach, E-HIPA [8] adopts an adaptive orthogonal matching pursuit (OMP) algorithm to reconstruct the sparse location vector. It can realize multi-target DFL without knowing the number of targets. Moreover, to mitigate the influence of the environmental dynamics in changing environments, DR-DFL [21] conducts joint location estimation and dictionary refinement based on the variational expectation-maximization (VEM) [22,23] algorithm.

DFL techniques estimate the locations of transceiver-free targets by analyzing their shadowing effects on wireless links. Received signal strength (RSS) [24] is one of the most widely used signatures of target locations, and the variance of RSS is commonly employed in DFL to characterize the shadowing effects caused by targets. As coarse-grained MAC-layer information, RSS readings are readily available in many commercial off-the-shelf (COTS) transceivers. It represents the overall signal power across all subcarriers of a wireless link. However, due to multipath fading and noises in changing environment [25], RSS readings are unreliable and vary with time. This has a negative effect on the localization performance of DFL. In view of the various drawbacks of RSS, channel state information (CSI) [26,27] has recently been leveraged for target localization. As fine-grained PHY-layer information, CSI represents the subcarrier-level channel measurements [28]. It can provide the signal strength and phase information of multiple channels.

In this paper, we propose to exploit the fine-grained subcarrier information for CS-based multi-target DFL in wireless sensor networks (WSN). To enrich the measurement information, we extract the signal features of CSI measurements from multiple channels. Then, the multi-target DFL is formulated as a joint sparse recovery problem. To solve this problem, an iterative location vector estimation algorithm is developed under the multitask Bayesian compressive sensing (MBCS) [29] framework. Subsequently, the location vector is estimated based on the recovered sparse vectors of multiple channels. Finally, we estimate the number and locations of multiple targets according to the estimated location vector.

Different from previous CS-based DFL methods, the proposed method leverages the frequency diversity of fine-grained subcarrier information to improve the localization accuracy. LCS and E-HIPA realize sparse reconstruction by greedy algorithms, while DR-DFL and the proposed method are based on Bayesian approaches. Moreover, conventional CS-based DFL methods estimate target locations by reconstructing a single sparse vector, while the proposed method needs to reconstruct multiple sparse vectors corresponding to multiple channels which share a joint sparsity pattern. Note that, the sparse reconstruction of each channel is not only based on its own measurements but also promoted by the measurement information from other channels. This offers the opportunity to adaptively borrow strength from the measurements of multiple channels to enhance the sparse recovery performance. In summary, the main contributions of this paper are as follows:

- We propose to use CSI measurements for CS-based DFL. Furthermore, we experimentally validate the feasibility of utilizing the fine-grained subcarrier information to improve the localization accuracy. To the best of our knowledge, this is the first work to leverage the frequency diversity of CSI measurements for CS-based DFL.
- We formulate the multi-target DFL as a joint sparse recovery problem, and develop a novel location vector estimation algorithm to solve this problem under the MBCS framework. The sparse reconstruction performance can be improved by sharing the measurement information among different channels.
- Target locations are estimated based on the recovered sparse vector of the channel that has the minimum residual error. We design a filter algorithm to mitigate the influence of the negligible but nonzero coefficients of the recovered sparse vector.

The rest of the paper is organized as follows: In Section 2, we review the related work of the DFL methods that use CSI. In Section 3, we give the signal model and formulate the multi-target DFL problem as a joint sparse recovery problem. The design and implementation of the proposed location vector estimation algorithm are illustrated in Section 4, and the simulation results are shown in Section 5. Finally, conclusions are given in Section 6.

## 2. Related Work

Pilot [30] is the first work that using CSI for DFL, which belongs to the fingerprinting-based DFL approaches. In Pilot, the correlation patterns of CSI measurements are regarded as fingerprints. Zhou et al. [31] proposed another fingerprinting-based DFL method to leverage CSI, which establishes a nonlinear relationship between CSI fingerprints and location information by support vector machine (SVM) regression. Gao et al. [32] construct a radio image based on CSI measurements and use the machine learning method to estimate the location of a target.

However, the fingerprinting-based DFL approaches involve site survey, which is labor-intensive and time-consuming, especially in the multi-channel case. To avoid this, Wang et al. [33] proposed a power fading model to characterize the relationship of CSI measurements and target location information. The target location can be estimated by solving a set of equations that are established based on the model. However, a dense deployment of wireless links is needed to ensure that the target is always sufficiently close to the line-of-sight (LOS) path of the wireless link. Moreover, Qian et al. [34] proposed a CSI-based tracking system. It can simultaneously estimate a target’s moving velocity and location based on a theoretical model, which geometrically quantifies the relationships between CSI measurements and the target location and velocity.

The aforementioned CSI-based DFL approaches focus on seeking appropriate location-dependent CSI features and trying to establish a robust and precise relationship between the CSI features and the location of a single target. How to make full use of the frequency diversity of CSI measurements to realize CS-based multi-target DFL is still a challenging issue. In this paper, to exploit the frequency diversity of the CSI measurements collected from multiple channels, we model the DFL problem as a joint sparse recovery problem and develop an iterative location vector estimation algorithm to solve this problem under the MBCS framework.

## 3. Problem Formulation

**Figure 1:**
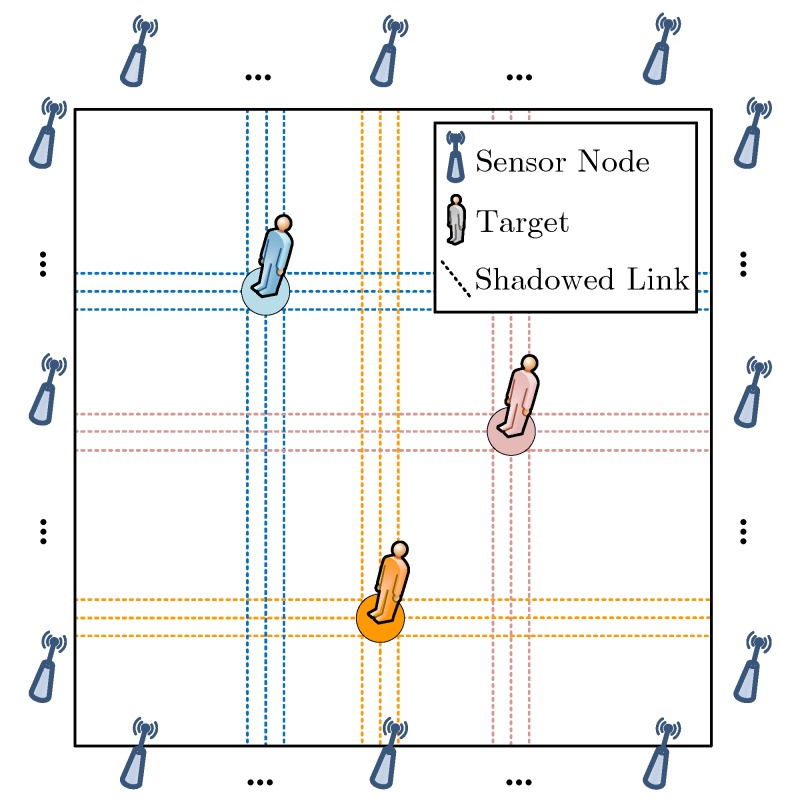
An illustration of CS-based multi-target DFL in WSN.

An illustration of the CS-based multi-target DFL in WSN is shown in Figure 1. The monitoring area is a two-dimensional (2D) (We consider the 2D location estimation in this paper. To estimate the 3D location of target, a shadowing model for 3D case is required. However, finding an accurate showing model for 3D case is still an open issue) rectangle region. *K* transceiver-free targets are randomly located in the region. To locate the targets, a WSN with *M* wireless links is deployed in the area of interest. Intuitively, targets in the monitoring area may inevitably shadow some radio signals of the wireless links, and the shadowing effects are closely related to the locations of targets. Therefore, by analyzing the target-induced shadowing effects, target location information can be inferred. Owing to the spatially distributed sparsity of targets, target location information can be considered as a sparse signal. We divide the monitoring area into *N* equal-sized grids, thus the target locations can be sparsely represented by

(1) $$s={\left[s_{1},s_{2},\ldots ,s_{n},\ldots ,s_{N}\right]}^{T},$$

where $s\in R^{N\times 1}$ denotes the location vector, $s_{n}\in \left\{0,1\right\}$ is its *n*-th element. If there exists a target in grid *n*, we set $s_{n}=1$; otherwise, $s_{n}=0$. Therefore, the locations of *K* targets can be denoted as $L=\left\{\left(x_{n},y_{n}\right)\left|s_{n}=1,n\in \left\{1,\ldots ,N\right\}\right.\right\}$, where $\left(x_{n},y_{n}\right)$ represents the Cartesian coordinate of the center of grid *n*.

As K≪N, s is a *K*-sparse vector. The main task of the CS-based multi-target DFL is to estimate s by analyzing the measurement information collected from *M* wireless links. In this paper, we propose to aggregate the fine-grained subcarrier information of multiple channels as the measurements and take advantage of their frequency diversity to improve the target counting and localization performance. As a fine-grained PHY-layer information, CSI provides the signal strength and phase information of multiple channels. It can be obtained from many COTS devices that implement an orthogonal frequency division multiplexing (OFDM) system [35,36]. We assume the CSI measurements are collected from *F* different channels. For link *m* and channel *f*, the CSI value is given as

(2) $$H_{m}^{f}=\left|H_{m}^{f}\right|e^{j\sin\{∠H_{m}^{f}\}},\;\;f=1,2,\ldots ,F,$$

where $H_{m}^{f}$ is a complex value, $∠H_{m}^{f}$ denotes the phase response, and $|H_{m}^{f}|$ denotes the amplitude response. It is noteworthy that $|H_{m}^{f}|$ is defined in linear (voltage) space, which represents the change of amplitude between the transmitter and receiver. By converting it into logarithmic (power) space, we can obtain the power fading with the unit of dB as [37]

(3) $${\tilde{H}}_{m}^{f}=20\log_{10}\left(|H_{m}^{f}|\right),$$

where ${\tilde{H}}_{m}^{f}$ denotes the power fading corresponding to link *m* and channel *f*. As discussed earlier, the target presented in the monitoring area will inevitably shadow some propagation paths of wireless signals. In this paper, we consider the change of power fading between the transmitter and receiver as the signal feature to characterize the target-induced shadowing effects. For link *m*, the change of power fading on channel *f* can be approximated as

(4) $$\Delta {\tilde{H}}_{m}^{f}={\tilde{H}}_{m}^{f}-{\tilde{H}}_{m}^{f}\left(0\right)\approx \varphi_{m}^{f}+\epsilon_{m}^{f},$$

where ${\tilde{H}}_{m}^{f}\left(0\right)$ denotes the reference power fading, which is recorded when the monitoring area is vacant. $\varphi_{m}^{f}$ represents the shadowing loss induced by the targets within the spatial impact area of link *m*. $\epsilon_{m}^{f}$ represents the measurement noise. Based on the collected CSI values, the power fading corresponding to *M* links and *F* channels can be obtained, which are written as

(5) $$Y=\left[y^{1},y^{2},\ldots ,y^{f},\ldots ,y^{F}\right]={\left[\begin{array}{cccc} y_{1}^{1} & y_{1}^{2} & \cdots & y_{1}^{F}\\ y_{2}^{1} & y_{2}^{2} & \cdots & y_{2}^{F}\\ \vdots & \vdots & \ddots & \vdots \\ y_{M}^{1} & y_{M}^{2} & \cdots & y_{M}^{F} \end{array}\right]}_{M\times F},$$

where $Y\in R^{M\times F}$ denotes the measurement matrix. Its *f*-th column $y^{f}\in R^{M\times 1}$ is a measurement vector corresponding to channel *f*. The *m*-th component of $y^{f}$ is $y_{m}^{f}$, which is equal to $\Delta {\tilde{H}}_{m}^{f}$. In the monitoring area, if any two targets are located sparsely [8], the shadowing loss $\varphi_{m}^{f}$ can be approximated as a summation of the attenuations that occur in the grids within the spatial impact area of link *m*. That is, the change of power fading can be expressed as the superposition of the perturbations caused by multiple targets, i.e.,

(6) $$y_{m}^{f}=\sum_{n=1}^{N}s_{n}\cdot \psi_{m,n}^{f}+\epsilon_{m}^{f},$$

where $\psi_{m,n}^{f}$ denotes the shadowing loss on link *m* and channel *f* that caused by a target in grid *n*. Based on Equation (6), the measurement vector corresponding to channel *f* is given as

(7) $$y^{f}=\Psi^{f}s+\epsilon^{f},$$

where $\epsilon^{f}\in R^{M\times 1}$ is a noise vector whose *m*-th component is $\epsilon_{m}^{f}$. $\Psi^{f}\in R^{M\times N}$ denotes the dictionary of channel *f* whose (m,n)-th element is $\psi_{m,n}^{f}$. According to the saddle surface model [38], $\psi_{m,n}^{f}$ can be described as

(8) $$\begin{array}{c} \psi_{m,n}^{f}=\gamma^{f}\cdot \left(\frac{1-\rho^{f}}{\lambda_{1}^{2}}U_{m,n}^{2}+\rho^{f}\cdot \left(1-\frac{V_{m,n}^{2}}{\lambda_{2}^{2}}\right)\right),\\ \;\;\;\;\;\;\;\;\;\;s.t.\;\;\;\;\frac{U_{m,n}^{2}}{\lambda_{1}^{2}}+\frac{V_{m,n}^{2}}{\lambda_{2}^{2}}⩽1, \end{array}$$

where $\gamma^{f}$ denotes the maximum shadowing loss on the LOS path, $\rho^{f}\in \left(0,1\right)$ denotes the shadowing ratio that is the normalized shadowing loss at the midpoint of the LOS path. Only the targets within the spatial impact area of link *m* can cause a nonzero $\psi_{m,n}^{f}$. Figure 2 depicts the spatial impact area of a wireless link, which is an ellipse between the transmitter and receiver. As shown in Figure 2, a *U*-*V* coordinate system is built in the spatial impact area. The midpoint of the LOS path is taken as the origin of the coordinates. The *U*-axis coincides with the LOS path, and the *V*-axis is vertical to it. In Equation (8), $\left(U_{m,n},V_{m,n}\right)$ denotes the coordinate of grid *n* under the *U*-*V* coordinate system of link *m*. $\lambda_{1}$ and $\lambda_{2}$ denote the semi-major and semi-minor axes of the elliptical spatial impact area, respectively.

As mentioned before, the target location vector s is a sparse vector. This means that s has only a few nonzero elements, i.e., ${∥s∥}_{0}=K$ (K≪N). Therefore, by taking advantage of the CS theory for accurate recovery of sparse signals, s can be reconstructed accurately with a few measurements (M≪N). In this case, Equation (7) is an under-determined linear system. This means that a unique solution for s may not exist. Taking into account the sparsity of s, recovering a unique solution for Equation (7) is possible. In particular, a sparsest solution can be obtained by solving the following $\ell_{0}$-norm minimization problem

(9) $$\begin{array}{c} \hat{s}=\arg\min_{s}{∥s∥}_{0},\\ \;\;s.t.\;∥y^{f}-\Psi^{f}{s∥}_{2}\leq \epsilon^{f}. \end{array}$$

However, in terms of computational complexity, directly solving the problem with $\ell_{0}$-norm is NP-hard [39]. The $\ell_{0}$-norm penalty encourages a maximally sparse solution, which is usually difficult to obtain. Fortunately, when $\Psi^{f}$ satisfies the RIP, Equation (9) can be approximated by the $\ell_{1}$-norm minimization that is polynomial-time solvable. LASSO [40] and basic pursuit (BP) [41] are well-known algorithms that realize sparse recovery via $\ell_{1}$-norm minimization. Using the linear programming technique to solve $\ell_{1}$-norm minimization problem and reconstruct the sparse vector is effective. However, it incurs considerable computational burden, especially in large-scale applications. Therefore, many greedy algorithms have been introduced to efficiently reconstruct the sparse signal. OMP [42] and GMP [20] are representative greedy algorithms. Furthermore, the problem could be solved in a Bayesian framework. Compared with the aforementioned linear programming and greedy algorithms, Bayesian compressed sensing (BCS) [43] recovers the sparse signal based on the posterior probability instead of a single value and could yield better sparse reconstruction performance.

In order to combat the negative effect of measurement noises and achieve better counting and localization performance, we propose to exploit the frequency diversity of the measurements collected from multiple channels and realize joint sparse recovery in DFL. For each channel, we set $\theta^{f}=\gamma^{f}\cdot s$ and $\phi_{m,n}^{f}=\psi_{m,n}^{f}/\gamma^{f}$. Then, the measurement model Equation (7) can be rewritten as

(10) $$y^{f}=\Phi^{f}\theta^{f}+\epsilon^{f},$$

where $\Phi^{f}$ is a dictionary whose (m,n)-th element is $\phi_{m,n}^{f}$. ${\left\{\theta^{f}\right\}}_{f=1}^{F}$ are unknown sparse vectors which share a common support with s. In this situation, the main task of target counting and localization is to reconstruct the jointly sparse vectors ${\left\{\theta^{f}\right\}}_{f=1}^{F}$ and find their common sparse supports.

## 4. Joint Sparse Recovery

**Figure 3:**
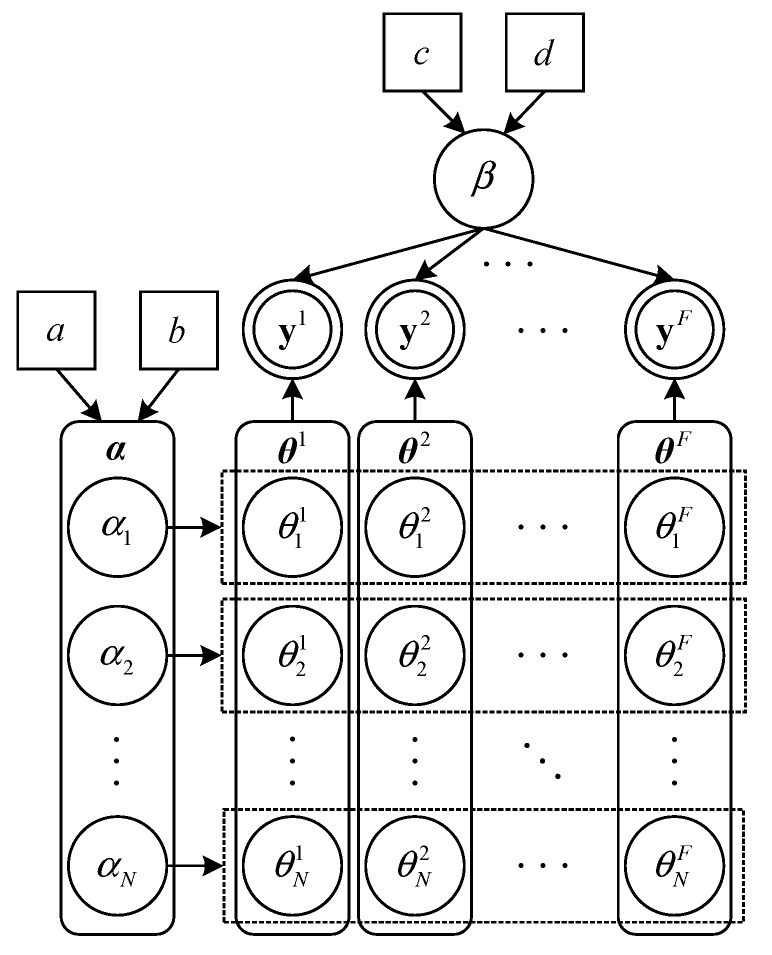
A hierarchical Bayesian model for joint sparse recovery.

In the previous section, we formulate the CS-based multi-target DFL as a joint sparse recovery problem. In this section, we approach this problem under the MBCS framework. A hierarchical Bayesian model for the joint sparse recovery is illustrated in Figure 3. The unknown sparse vectors ${\left\{\theta^{f}\right\}}_{f=1}^{F}$ are regarded as stochastic variables, and a non-separable sparsity inducing prior model is imposed on them [44]. It is a two-layer hierarchical prior model. In the first layer, we assume $\theta^{f}$ follows a zero-mean Gaussian prior distribution

(11) p(θfα)=∏n=1NNθnf0,αn−1=2π−N−N22Λ12exp12(θf)TΛθf,

where $\alpha_{n}$ denotes the inverse variance (precision) of ${\left\{\theta_{n}^{f}\right\}}_{f=1}^{F}$, $\alpha ={\left[\alpha_{1},\alpha_{2},\ldots ,\alpha_{N}\right]}^{T}$, and $\Lambda =diag\left(\alpha \right)$. As shown in Figure 3, ${\left\{\alpha_{n}\right\}}_{n=1}^{N}$ are common hyperparameters of ${\left\{\theta^{f}\right\}}_{f=1}^{F}$. In the second layer of the prior model, we impose the following Gamma distribution on α since Gamma distribution is conjugate to Gaussian distribution

(12) $$p\left(\alpha ;a,b\right)=\prod_{n=1}^{N}\mathrm{Gamma}\left(\alpha_{n}\left|a,b\right.\right)=\prod_{n=1}^{N}\frac{1}{\Gamma \left(a\right)}b^{a}\alpha_{n}^{a-1}\exp\left(-b\alpha_{n}\right),$$

where $\mathrm{Gamma}\left(\cdot \left|a,b\right.\right)$ is a Gamma distribution with parameters *a* and *b*. $\Gamma \left(a\right)=\int_{0}^{\infty }x^{a-1}e^{-x}dx$ represents the Gamma function. The proposed hierarchical prior model is used to promote the joint sparsity of the sparse vectors ${\left\{\theta^{f}\right\}}_{f=1}^{F}$. It can achieve efficient information-sharing between channels. The measurements from all channels contribute to the learning of the common hyperparameters, thus making it possible to transfer information between channels. In particular, the sparse reconstruction of each individual channel is affected by both its own measurements and the measurement information from other channels. This offers the opportunity to adaptively borrow strength from the measurements of different channels to enhance the sparse recovery performance.

The measurement noise $\epsilon^{f}$ is considered as a zero-mean Gaussian random variable, and the inverse variance of $\epsilon_{m}^{f}$ is assumed to be β. Therefore, the likelihood function for $\theta^{f}$ can be written as

(13) $$p\left(y^{f}|\theta^{f},\beta \right)={\left(\frac{2\pi }{\beta }\right)}^{-\frac{M}{2}}\exp\left(-\frac{\beta }{2}{∥y^{f}-\Phi^{f}\theta^{f}∥}_{2}^{2}\right).$$

A Gamma distribution is imposed on β, i.e.,

(14) $$p\left(\beta^{f};c,d\right)=\mathrm{Gamma}\left(\beta |c,d\right)=\frac{1}{\Gamma (c)}d^{c}\beta^{c-1}\exp\left(-d\beta \right),$$

where *c* and *d* are deterministic parameters. The Gamma distribution is chosen since it is conjugate to the Gaussian likelihood, and thus the associated Bayesian inference can be performed in closed form [23]. The posterior distribution for $\theta^{f}$ can be derived as

(15) $$p\left(\theta^{f}|y^{f},\alpha ,\beta \right)=\frac{p\left(y^{f}|\theta^{f},\beta \right)p\left(\theta^{f}|\alpha \right)}{\int p\left(y^{f}|\theta^{f},\beta \right)p\left(\theta^{f}|\alpha \right)d\theta^{f}}=N\left(\theta^{f}|\mu^{f},\Sigma^{f}\right),$$

with mean vector $\mu^{f}$ and covariance matrix $\Sigma^{f}$ given by

(16) $$\mu^{f}=\beta \Sigma^{f}{\left(\Phi^{f}\right)}^{T}y^{f},$$

and

(17) $$\Sigma^{f}={\left(\beta {\left(\Phi^{f}\right)}^{T}\Phi^{f}+\Lambda \right)}^{-1}.$$

Note that, Equation (17) contains matrix inversion operation. To obtain ${\left\{\Sigma^{f}\right\}}_{f=1}^{F}$, the computational complexity is $O\left(N^{3}F\right)$. Using the matrix inversion lemma [45], $\Sigma^{f}$ can be evaluated as

(18) $$\Sigma^{f}=\Lambda^{-1}-\Lambda^{-1}{\left(\Phi^{f}\right)}^{T}{\left(\Theta^{f}\right)}^{-1}\Phi^{f}\Lambda^{-1},$$

where

(19) $$\Theta^{f}=\Phi^{f}\Lambda^{-1}{\left(\Phi^{f}\right)}^{T}+\beta^{-1}I_{M}.$$

With the above matrix transformation, we only need to compute the inversion of $\Theta^{f}$. Because $\Theta^{f}$ is an M×M matrix, the computational complexity can be reduced to $O\left(M^{3}F\right)$. In addition, Λ is an N×N diagonal matrix, and its inversion is easy to obtain.

Subsequently, we have to estimate the common hyperparameters α and β. The estimation can be derived by maximizing the following logarithm marginal likelihood

(20) $$\begin{array}{cc} L(\alpha ,\beta )= & \sum_{f=1}^{F}\log p(y^{f}|\alpha ,\beta )\\ = & \sum_{f=1}^{F}\log p\left(y^{f}|\theta^{f},\beta \right)p\left(\theta^{f}|\alpha \right)d\theta^{f}\\ = & -\frac{1}{2}\sum_{f=1}^{F}\left(M\log2\pi +\log|\Theta^{f}|+{\left(y^{f}\right)}^{T}{\left(\Theta^{f}\right)}^{-1}y^{f}\right). \end{array}$$

Differentiating L(α,β) with respect to $\alpha_{n}$, and setting the result to zero and rearranging, yields

(21) $$\alpha_{n}^{New}=\frac{F-\alpha_{n}\sum_{f=1}^{F}\Sigma_{n,n}^{f}}{\sum_{f=1}^{F}{\left(\mu_{n}^{f}\right)}^{2}},$$

where $\alpha_{n}^{New}$ denotes the updated hyperparameter, $\Sigma_{n,n}^{f}$ is the (n,n)-th element of $\Sigma^{f}$, and $\mu_{n}^{f}$ is the *n*-th component of $\mu^{f}$. Next, differentiate L(α,β) with respect to β, and then set the results to zero, yielding

(22) $$\beta^{New}=\frac{\sum_{f=1}^{F}\left(M-N+\sum_{n=1}^{N}\alpha_{n}\Sigma_{n,n}^{f}\right)}{\sum_{f=1}^{F}{∥y^{f}-\Phi^{f}\mu^{f}∥}_{2}^{2}}.$$

Based on the above update rules, we develop a novel iterative location vector estimation algorithm, which iterates between Equations (16)–(19) and Equations (21) and (22), until a convergence criterion has been satisfied. The procedure of location vector estimation is specified in Algorithm 1. We set the convergence criterion of the iterative procedure as the second norm of the residual error γ smaller than a threshold $\gamma_{\mathrm{th}}$, or the iteration number τ has reached a maximal allowable value $\tau_{\max}$.

After estimating the posterior distributions of the sparse vectors ${\left\{\theta^{f}\right\}}_{f=1}^{F}$, their common support can be inferred from the mean vectors ${\left\{\mu^{f}\right\}}_{f=1}^{F}$. In Algorithm 1, we estimate the location vector s based on $\mu^{\hat{f}}$, where $\hat{f}$ denotes the index of the channel that achieves the minimum residual error. However, $\mu^{\hat{f}}$ may not be strictly sparse. It usually contains many negligible but nonzero coefficients. To mitigate the effect of small coefficients, we filter out them with a sparsity threshold $\eta_{\mathrm{th}}$, which is shown in Line 9 of Algorithm 1. When we obtain the estimated location vector $\hat{s}$, the target number can be estimated as $\hat{K}={∥\hat{s}∥}_{0}$, and the estimated target locations are given as $\hat{L}=\left\{\left(x_{n},y_{n}\right)|{\hat{s}}_{n}=1,n\in \left\{1,\ldots ,N\right\}\right\}$.

**Algorithm 1** Location Vector Estimation1: **Initialization:**2: $\gamma_{\mathrm{th}}=10^{-3}$, $\tau_{\max}=10^{3}$, $\eta_{\mathrm{th}}=-10\mathrm{dB}$, γ=τ=0.3: **while** ($\gamma ⩾\gamma_{\mathrm{th}}$ or $\tau ⩽\tau_{\max}$) **do**4:  Calculate $\mu^{f}$ and $\Sigma^{f}$ by using Equation (16)–(19).5:  Update α and β by using Equation (21)–(22).6:  $\gamma \leftarrow \sum_{f=1}^{F}{∥y^{f}-\Phi^{f}\mu^{f}∥}_{2},\tau \leftarrow \tau +1$.7: **end while**8: Choose $\hat{f}$ that minimizes $∥y^{f}-\Phi^{f}\mu^{f}{∥}_{2}$.9: If 20lgμnf^/μnf^maxiμif^maxiμif^<ηth, set $\mu_{n}^{\hat{f}}=0$; otherwise, $\mu_{n}^{\hat{f}}=1$.10: Let the estimated location vector $\hat{s}=\mu^{\hat{f}}$.

## 5. Simulation Results

### 5.1. Simulation Setup

In this section, a series of numerical simulations are conducted to illustrate the performance of the proposed scheme. The simulations are carried out in MATLAB R2015b 64bit version running on a PC with i7-8550U CPU and 16 GB memory. We consider an l×l square region as the monitoring area. It has been divided into *N* equal-sized grids, and *K* targets are randomly located in it. Sensor nodes are uniformly deployed along the perimeter of the monitoring area, and each node is placed at the midpoint of a grid side. They can transmit and receive radio signals on *F* channels, and we establish *M* bidirectional wireless links between them. In real environments, it is inevitable for measurements to be corrupted with noises. In our simulation, to validate the reliability and robustness of DFL methods, we intentionally add zero-mean white Gaussian noises to the measurements of multiple channels. For channel *f*, the signal-to-noise ratio (SNR) of the measurements $y^{f}$ is defined as 10lg(∥Ψfs∥22/yf22(Mσf)(Mσf)), where $\sigma^{f}$ represents the variance of $\epsilon^{f}$. Table 1 gives the default values of some simulation parameters.

In our simulations, all results are averaged over *T* Monte Carlo trials. When the channel number is large, the proposed method is time-consuming. Therefore, if we set *T* to be a very large value, the overall simulation time will be extremely long. In view of this, we set T=500 to balance the simulation time and the accuracy of results. In addition, it should be noted that, in real deployment, the localization algorithm is conducted on a server that for data processing and analysis. For each trial, the localization error $E_{t}$ is computed as

(23) $$E_{t}=\frac{\sum_{k=1}^{K}\sqrt{{\left({\hat{x}}^{k}-x^{k}\right)}^{2}+{\left({\hat{y}}^{k}-y^{k}\right)}^{2}}}{K},$$

where $\left(x^{k},y^{k}\right)$ and $\left({\hat{x}}^{k},{\hat{y}}^{k}\right)$ denote the ground-truth location and estimated location of target *k*, respectively. There are $\min\{K,\hat{K}\}$ pairs of $\left\{\left(x^{k},y^{k}\right),\left({\hat{x}}^{k},{\hat{y}}^{k}\right)\right\}$, and we need to find the optimal values for $\left(x^{k},y^{k}\right)$ and $\left({\hat{x}}^{k},{\hat{y}}^{k}\right)$ from L and $\hat{L}$, respectively. Intuitively, there are $\left(\max\left\{K,\hat{K}\right\}\right)!/(|K-\hat{K}\left|\right)!$ different conditions of the pairs, and we choose the condition that achieves the minimum $E_{t}$ as the matching result. To evaluate the counting and localization performance of the multi-target DFL, we define the following three metrics:

- Average localization error ($E_{A}$), which denotes the average Euclidean distance between the true and estimated target locations, i.e.,
  (24) $$E_{A}=\frac{\sum_{t=1}^{T}E_{t}}{T}.$$
- Root-mean-square localization error ($E_{R}$), which is defined as
  (25) $$E_{R}=\sqrt{\frac{\sum_{t=1}^{T}E_{t}^{2}}{T}}.$$
- Correct counting rate ($P_{C}$), which denotes the probability of correctly estimating the target number (i.e., $\hat{K}=K$).

### 5.2. Impact of the Number of Iterations

**Figure 4:**
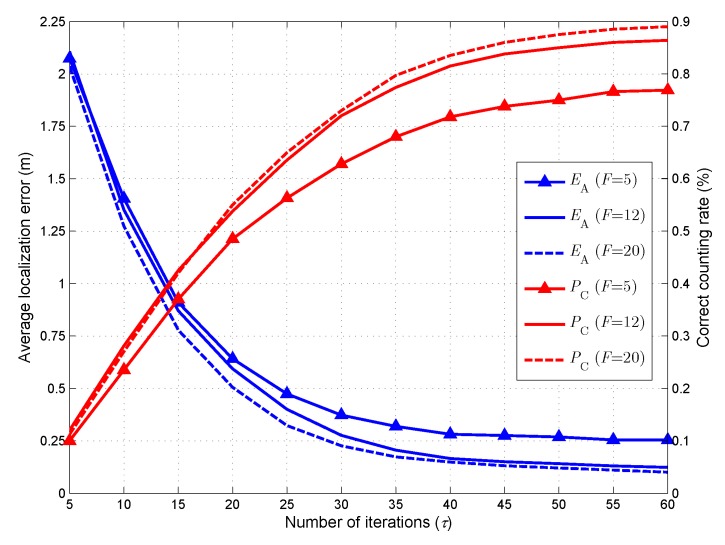
Counting and localization performance when the number of iterations varies from 5 to 60.

In the first simulation, we investigate the impact of the iteration number τ on the target counting and localization performance. In Section IV, an iterative location vector estimation algorithm is proposed to solve the joint sparse recovery problem. Intuitively, the estimation accuracy of the algorithm is closely related to the number of iterations. To reveal the relationship between the iteration number and the estimation performance, we test $E_{A}$ and $P_{C}$ of the proposed method when τ varies from 5 to 60. The results are shown in Figure 4. As seen, with the increasing of τ, $E_{A}$ decreases and $P_{C}$ increases accordingly. We also compare the performance of the proposed method when F=5,12, and 20. It can be observed from Figure 4 that increasing the number of channels can result in a more accurate estimation of the target number and locations. It should be noted that although we can achieve a better performance with a larger value of τ, it may result in heavy computational load. For this reason, we set $\tau_{\max}=50$ as a tradeoff between accuracy and computational cost.

### 5.3. Impact of the Number of Channels

**Figure 5:**
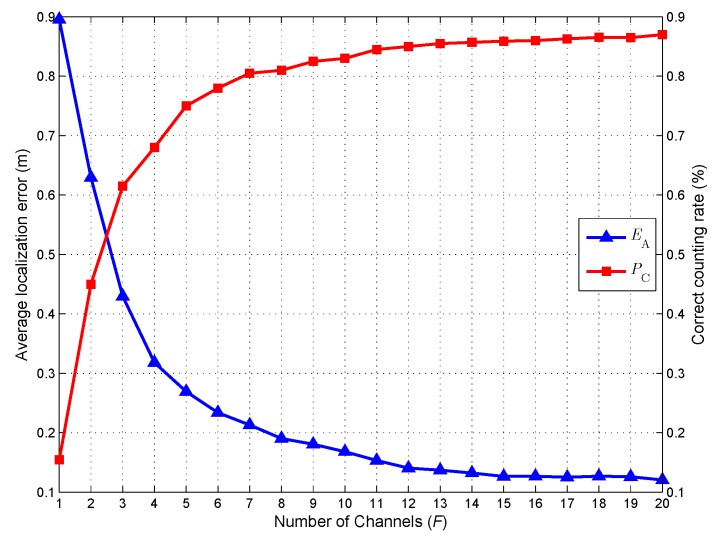
Counting and localization performance when the number of channels varies from 1 to 20.

In the second simulation, we test the effect of the channel number *F* on the target counting and localization performance of the proposed method. Figure 5 shows the average localization error $E_{A}$ and correct counting rate $P_{C}$ as functions of the channel number *F*. The figure illustrates that increasing *F* has a positive effect on both quantities. This trend is consistent with the fact that increasing the number of channels, more useful information will be provided for the target counting and localization. The simulation results confirm the effectiveness of the proposed method for exploiting the frequency diversity of CSI measurements. With the increase of *F*, $E_{A}$ decreases and $P_{C}$ increases accordingly. However, when F>12, very little performance improvement can be obtained by increasing the channel number *F*. In fact, a large *F* means a complex Bayesian model and the posteriors of more stochastic variables need to be inferred. For this analysis, an excessively large *F* may have a negative effect on the accuracy of the posterior inference. When F>12, the negative effect of increasing the complexity of the Bayesian model will almost offset the positive effect contributed by increasing the frequency diversity of CSI measurements. In view of this, we choose the channel number F=12 as our default setting.

### 5.4. Comparison of Localization Accuracies for Different DFL Methods

**Figure 6:**
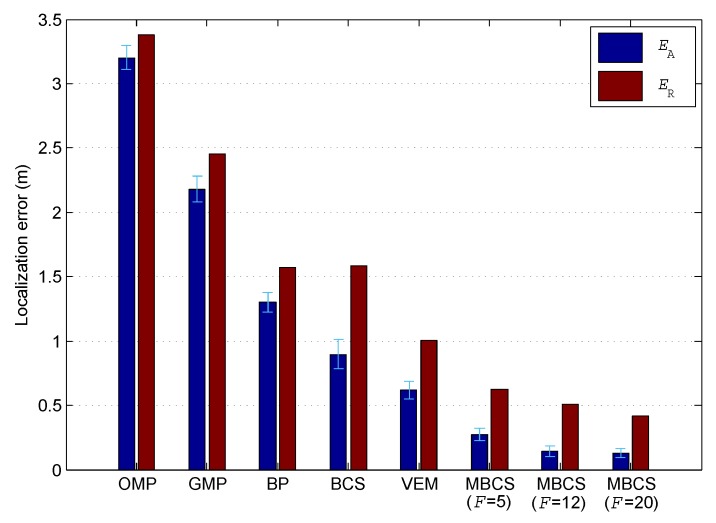
Comparison of localization accuracies for different DFL methods.

In the following, we compare the localization accuracies of the proposed method and the CS-based multi-target DFL approaches that using the following sparse recovery algorithms: OMP, BP, GMP, BCS, and VEM (these abbreviations are explained at the end of the article). Figure 6 depicts the average localization error $E_{A}$ and root-mean-square localization error $E_{R}$ of these DFL approaches. The 95% confidence interval of $E_{A}$ is also shown in this figure. As can be seen, the proposed method significantly outperforms all the other approaches in terms of both $E_{A}$ and $E_{R}$. Furthermore, we can also see that the Bayesian methods (i.e., BCS, VEM, and MBCS) can achieve a better performance than other methods. Particularly, although the improvement on $E_{A}$ of the proposed method is only 0.0135 m by increasing the channel number *F* from 12 to 20, the corresponding reduction in $E_{R}$ is 0.0909 m. This indicates that the increase in the number of channels can lead to a more robust localization performance.

### 5.5. Localization Error vs. Number of Targets

**Figure 7:**
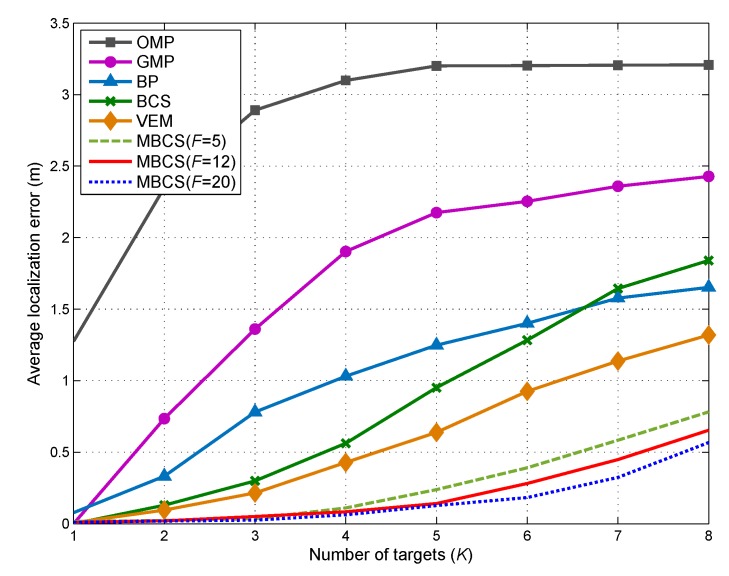
Impact of target number on average localization error.

Next, we examine the localization performances of multiple DFL approaches under different number of targets. Figure 7 shows the comparison of the average localization error $E_{A}$ versus the number of targets *K*. As expected, $E_{A}$ is an increasing function of *K*. When *K* increases from 1 to 8, the average localization errors for all DFL methods increase dramatically. This is because, with the increase in *K*, the sparsity level of location vector and the joint sparsity level of ${\left\{\theta^{f}\right\}}_{f=1}^{F}$ will decease accordingly. In this case, the reconstruction accuracy of the location vector will be degraded according to the principle of the CS theory. Furthermore, owing to the ability of exploiting the frequency diversity of the CSI measurements, the proposed method can achieve the lowest $E_{A}$ among all the other methods. Additionally, it is observed that the localization performance of the proposed method will be improved when *F* increases. This indicates that, under the same sparsity level, the increase of channel number can enrich the measurement information and lead to more accurate localization.

### 5.6. Localization Error vs. SNR

**Figure 8:**
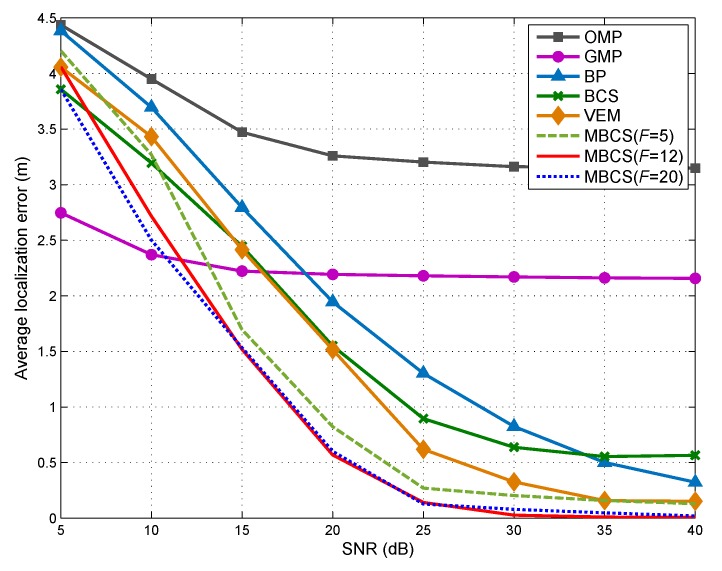
Impact of SNR on average localization error.

In the last simulation, we turn our attention to the impact of SNR on the localization accuracies of multiple DFL approaches. Figure 8 plots the average localization error $E_{A}$ versus SNR. As SNR increases from 5dB to 40dB, the average localization errors of all six DFL approaches experience a significant drop. When SNR<25 dB, the localization performance of the proposed method is drastically affected by the noise increase. It can also be observed that the proposed method outperforms other DFL methods in most cases (SNR>13.2 dB). Particularly, in the case where the SNR is high (e.g., SNR=30 dB), we notice that the proposed method leads to improvements in terms of $E_{A}$ are 0.798 m and 0.299 m over the BP-based DFL and VEM-based DFL, respectively.

## 6. Conclusions

In this paper, we presented a novel CS-based multi-target DFL method, which exploits the fine-grained subcarrier information for target counting and localization in WSN. The key novelty of the proposed method is the making use of the frequency diversity of CSI measurements for the CS-based multi-target DFL. We formulate the multi-target DFL as a joint sparse recovery problem, and adopt an MBCS-based algorithm to simultaneously reconstruct the sparse vectors of multiple channels. The target counting and localization performance can be improved with the assistance of the frequency diversity. To validate the merits of the proposed method, we perform an extensive simulation study to compare with the state-of-the-art CS-based multi-target DFL approaches. Simulation results confirmed the effectiveness and robustness of the proposed method.

## Figures and Tables

**Figure 2:**
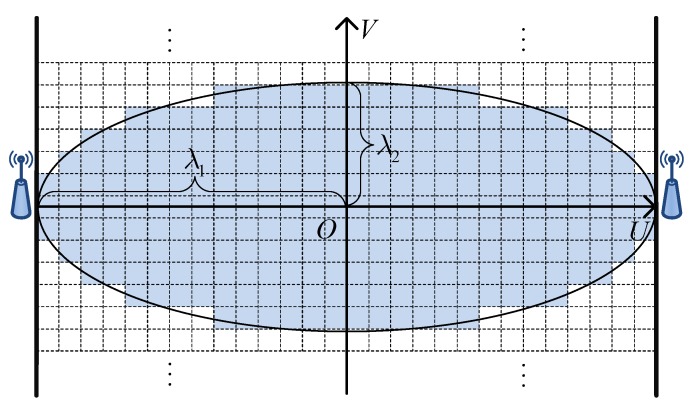
Spatial impact area of a wireless link.

**Table 1:** Default values of simulation parameters.

Parameters	Explains	Default Values
*l*	length of wireless link	14 m
$\lambda_{1}$	semi-major axis	7 m
$\lambda_{2}$	semi-minor axis	2.75 m
*N*	number of grids	784
*M*	number of wireless links	56
*K*	number of targets	5
*F*	number of channels	12
SNR	signal-to-noise ratio	25 dB
τ	number of iterations	50

## Abbreviations

The following abbreviations are used in this manuscript:

DFL	Device-free localization
CS	Compressive sensing
MBCS	Multitask Bayesian compressive sensing
LBS	Location-based service
RF	Radio frequency
RTI	Radio tomographic imaging
CT	Computed tomography
SLMLU	Sparse localization approach with a mobile beacon based on LU decomposition
RIP	Restricted isometry property
LCS	Device-free localization based on compressive sensing
GMP	Greedy matching pursuit
E-HIPA	The energy-efficient framework for high-precision multi-target-adaptive device-free localization
OMP	Orthogonal matching pursuit
DR-DFL	Dictionary refinement based device-free localization
VEM	Variational expectation-maximization
RSS	Received signal strength
COTS	Commercial off-the-shelf
CSI	Channel state information
WSN	Wireless sensor networks
SVM	Support vector machine
LOS	Line-of-sight
OFDM	Orthogonal frequency division multiplexing
LASSO	Least absolute shrinkage and selection operator
BP	Basic pursuit
BCS	Bayesian compressive sensing
SNR	Signal-to-noise ratio
